# A sensitive method for analyzing fluconazole in extremely small volumes of neonatal serum

**DOI:** 10.1186/s40780-020-00170-y

**Published:** 2020-07-01

**Authors:** Jumpei Saito, Ayano Tanzawa, Yuka Kojo, Hidehiko Maruyama, Tetsuya Isayama, Kensuke Shoji, Yushi Ito, Akimasa Yamatani

**Affiliations:** 1 Department of Pharmacy, National Center for Child Health and Development, 157-8535, 2-10-1 Okura, Setagaya-ku, Tokyo, Japan; 2 Division of Neonatology, Center for Maternal-Fetal, Neonatal and Reproductive Medicine, National Center for Child Health and Development, Tokyo, Japan; 3 Division of Infectious Diseases, Department of Medical Subspecialties, National Center for Child Health and Development, Tokyo, Japan

**Keywords:** Fosfluconazole, Fluconazole, Liquid chromatography-tandem mass spectrometry, Neonate, Serum sample volume

## Abstract

**Background:**

The need for a large volume of serum sample significantly reduces the feasibility of neonatal pharmacokinetic studies in daily practice, which must often rely on scavenged or opportunistic sampling. This problem is most apparent in preterm newborns, where ethical and practical considerations prohibit the collection of large sample volumes. Most of the fluconazole analysis assays published thus far required a minimum serum sample of 50 to 100 μL for a single assay. The purpose of the present study was to develop and validate a sensitive method requiring a smaller sample volume (10 μL) to satisfy clinically relevant research requirements.

**Methods:**

Following simple protein precipitation and centrifugation, the filtrated supernatant was injected into a liquid chromatography system and separated with a C18 reverse-phase column. Fluconazole and the internal standard (IS, fluconazole-d4) were detected and quantified using tandem mass spectrometry. The method was validated with reference to the Food and Drug Administration’s Guidance for Industry. Accuracy and precision were evaluated at six quality control concentration levels (ranging from 0.01 to 100 μg/mL).

**Results:**

Investigated calibration curves were linear in the 0.01–100 μg/mL range. Intra- and inter-day accuracy (− 7.7 to 7.4%) and precision (0.3 to 6.0%) were below 15%. The calculated limit of detection and the lower limit of quantification (LLOQ) was 0.0019 μg/mL and 0.0031 μg/mL, respectively. Fluconazole in the prepared samples was stable for at least 4 months at − 20 °C and − 80 °C. This method was applied to analyze 234 serum samples from ten neonates who received fosfluconazole, a water-soluble phosphate prodrug of fluconazole which converts to fluconazole in the body, as part of a pharmacokinetic study using daily scavenged laboratory samples. The median (range) concentration up to 72 h after fosfluconazole administration was 2.9 (0.02 to 26.8 μg/mL) μg/mL, which was within the range of the calibration curve.

**Conclusion:**

Fluconazole was able to be detected in an extremely small volume (10 μL) of serum from neonates receiving fosfluconazole. The method presented here can be used to quantify fluconazole concentrations for pharmacokinetic studies of the neonatal population by using scavenged samples.

## Background

The incidence of invasive fungal infection ranges from 2 to 16% in neonates with very low birth weight (VLBW < 1500 g) [1, 2], and a higher incidence was found in infants with lower birth weight and in premature neonates [3]. In previous studies, the mortality rate following *Candida* infection was found to range from 30 to 40%, and neurodevelopmental impairment was common among survivors [4, 5]. Although the universal use of antifungal prophylaxis is still controversial, prophylactic fluconazole is generally recommended for extremely low birth weight (ELBW < 1000 g) neonates, especially in neonatal intensive care units (NICU) with a prevalence of *Candida spp.* infection higher than 5% [6]. At our center, fosfluconazole, a water-soluble phosphate prodrug of fluconazole which is more highly soluble than fluconazole, is frequently used for antifungal prophylaxis in preterm neonates to minimize the infusion volume [7]. Although the pharmacokinetics of fluconazole after fosfluconazole treatment in adults is well documented, an appropriate, validated dosing regimen for neonates with VLBW and ELBW does not yet exist, and further clinical pharmacokinetic (PK) research is required. However, to be able detect fluconazole at a quantification limit of 0.5 μg/mL for PK studies [8], a 100 to 200 μL whole blood sample (about 50 to 100 μL of plasma or serum) is required for a single assay (Table 1) [9–24]. To perform replicate analysis to reduce the preparation bias, two- to three-fold this quantity is needed [25]. However, in preterm neonates, blood sampling is limited due to ethical and practical considerations. Although an opportunistic strategy using scavenged samples combined with population PK modeling has been proposed as an alternative to conventional methods of performing PK studies in neonates, the need for a large sample volume significantly limits its feasibility.

**Table 1:** Existing methods for fluconazole quantification in human whole blood, plasma, and serum

	Method	Sample type	Sample volume (μL)	Sample preparation	LOQ (μg/mL)	Reference No.
1	LC-MS	Serum	25	No pretreatment	0.1	[9]
2	LC-MS	Plasma	50	LLE	0.0005	[10]
3	LC-MS	Plasma	50	PP	0.03	[11]
4	LC-UV	Serum	50	SPE	0.1	[12]
5	LC-UV	Plasma	50	SPE	0.2	[13]
6	LC-MS	Plasma	70	PP	0.01	[14]
7	LC-MS	Serum	75	PP	0.06	[15]
8	LC-MS	Plasma	100	PP	0.1	[16]
9	LC-MS	Plasma	100	PP	0.1	[17]
10	LC-MS	Plasma	100	PP	0.1	[18]
11	LC-MS	Serum	100	PP	0.2	[19]
12	LC-MS	Plasma	100	DBS	0.5	[20]
13	LC-MS	Serum	100	PP	0.5	[21]
14	LC-UV	Plasma	300	SPE	0.05	[22]
15	LC-UV	Whole blood	300	SPE	0.5	[23]
16	LC-UV	Plasma	500	LLE	0.4	[24]

The reported fluconazole determination method has a limit of quantification equal to or greater than 0.5 μg/mL

*Abbreviations: LC* liquid chromatography, *MS* mass-spectrometry, *UV* ultraviolet, *PP* protein precipitation, *LLE* liquid-liquid extraction, *SPE* solid phase extraction, *DBS* dried blood spot

## Methods

### Chemicals and standards

Fluconazole (> 99.0%) and its internal standard (IS, fluconazole-d4 stable isotope (> 99.0%)) were purchased from Toronto Research Chemical (North York, Ontario, Canada). Mass spectrometry-grade methanol, acetonitrile, formic acid, and ammonium formate were obtained from Sigma-Aldrich (St. Louis, MO, USA). Ultra-pure water was obtained using a Milli-Q system (Millipore, Bedford, USA). Stock solutions of fluconazole (2 mg/mL) and IS (1 mg/mL) were prepared in acetonitrile and stored at − 80 °C in a freezer.

### Liquid chromatography-tandem mass spectrometry conditions

The liquid chromatography-tandem mass spectrometry (LC-MS/MS) system used in the present study consisted of the TSQ Vantage triple stage quadrupole mass spectrometer connected to Dionex UltiMate 3000 (Thermo Fisher Scientific K.K., Tokyo, Japan). The LC system consisted of an Ultimate 2000 SRD degasser, Ultimate 3000 RS binary solvent pump system, column oven, and Ultimate 3000 RS autosampler. Identification of the compound was based on the relative retention time and product ion ratio. Chromatographic separation of fluconazole and IS was performed over a total run time of 5 min on the Imtakt^®^ UK-C18 column (3 μm, 3.0 mm × 50 mm, Imtakt, Kyoto, Japan) and was maintained at 37 °C. The mobile phases consisted of 0.1% formic acid and 10 mM ammonium formate in water (pH 3.0) (mobile phase A) and 0.1% formic acid and 10 mM ammonium formate in methanol (mobile phase B). A linear gradient was run at a flow rate of 0.4 mL/min. Mobile phase B was run at 5% over 0–1 min, 5–95% over 1–2 min, maintained at 95% for 1 min (3–4 min), then returned to 5% (initial condition). The samples were kept at 10 °C in the auto-sampler, and a 10 μL volume was injected into the LC system.

The LC-MS/MS conditions were as follows: Electrospray ionization analysis was conducted in the positive mode; the spray voltage was 3500 V; the vaporizer temperature was 400 °C; the sheath gas and auxiliary gas (both nitrogen) pressure was 60 and 10 arbitrary units, respectively; the in-source collision-induced dissociation was set at 4 V; and the capillary temperature was 350 °C. The collision energy and S-lens voltage were set at 15 eV and 70 V for fluconazole and 13 eV and 74 V for IS. The collision gas (argon) pressure at the second quadrupole was 1.5 mTorr. Mass spectra were acquired by selected reaction monitoring. The optimal MS/MS transitions were determined separately by direct infusion of each compound solution at a 10 μL/min flow rate into the MS/MS detector at a 10 μg/mL concentration in mobile-phase mixing. The selected m/z transitions were 307.1 > 238.1 for fluconazole and 311.1 > 242.2 for IS. Chromatographic data acquisition, peak integration, and quantification were performed using the QUAL and QUAN browsers of the Xcalibur software package (Thermo Fischer Scientific K.K., Tokyo, Japan).

### Sample preparation

For sample preparation, 4 μL IS (25 μg/mL in acetonitrile) and 40 μL acetonitrile were added to each 10 μL serum sample. Subsequently, the samples were vortexed for 30 s before being centrifuged (11,180×*g*) at 4 °C for 10 min. Approximately 35 to 40 μL of supernatant was transferred to a 0.2 μm filtration tube (Nacalai Tesque, Inc., Kyoto, Japan). Samples obtained by filtration were centrifuged (1000×*g*) at 4 °C for 1 min before being transferred into auto-sampler vials.

### Calibration standards and quality control

A calibration standard curve for fluconazole was constructed by preparing six blank serum samples ranging in quantity from 0.01 to 100 μg/mL (0.01, 0.1, 1, 10, 50 and 100 μg/mL). These samples were also used for quality control (QC) and were stored at − 80 °C.

### Limit of detection (LOD) and lower limit of quantification (LLOQ)

The limit of detection (LOD) was defined as the lowest concentration at which the analyte was able to be detected reliably, and the lower limit of quantification (LLOQ) was defined as the lowest concentration at which the analyte was able to be quantified, according to the Food and Drug Administration [26]. The LOD was determined on the basis of the standard deviation of the response (Sy) of the curve and the slope of the calibration curve (S) [27]. The LOD was approximately 3.29 (Sy/S). The standard deviation of the response was determined on the basis of the standard deviation of the y-intercept of the regression line. For the LLOQ, the calculation method was again based on the Sy and S according to the formula: LLOQ = 10 (Sy/S).

### Recovery, precision, and accuracy

Precipitation recovery was determined by calculating the ratio (%) of the area response of the samples (normalized by the internal standard) after precipitation with the pure standard solutions at QC concentrations. Accuracy was determined by replicate analyses of the six concentrations (0.01, 0.1, 1, 10, 50, and 100 μg/mL). The deviation of the mean from the true value was expected to be within 15% of the actual value [26]. The precision of the assay was measured as the percent coefficient of variation for the eight concentrations during the course of validation. Intraday assay variations were determined by analyzing six replicate samples at each concentration on a single day. Interday assay variations were determined by analyzing four replicate samples at each concentration on four separate days.

### Matrix and carry-over effects

Matrix effect was checked with six different lots of serum. Three replicate samples each of the two concentration samples (0.1 and 100 μg/mL) were prepared from different lots of plasma (36 QC samples in total). Potential sample carry-over was assessed by analyzing serum samples spiked with concentrations of fluconazole at the highest QC sample (100 μg/mL) followed by blank samples. The carry-over test was repeated six times.

### Stability

A series of serum samples spiked with fluconazole at concentrations of 0.01, 0.1, 1, 10 and 100 μg/mL were prepared, and the final samples were stored at − 20 °C and − 80 °C for 4 months. For each concentration, three replicate samples were analyzed. The stability of fluconazole in five QC samples was studied under the autosampler condition of 4 °C for 48 h by comparing the peak areas obtained from the stored samples and freshly prepared samples. For freeze-thaw stability test, fluconazole concentrations in five QC samples after six freeze-thaw cycles were evaluated. The samples were considered stable if the difference in peak area between the stored and freshly prepared samples was ≤15%.

### Application

This method was developed to enable fluconazole PK studies in preterm neonates and was approved by the ethical board of the National Center for Child Health and Development. The following were the inclusion criteria: prescription of intravenous fosfluconazole (Prodif® intravenous solution 100 mg/1.25 mL, Pfizer) for prophylaxis against fungal infection and neonates admitted to the neonatal intensive care unit. Patients were only recruited after written informed consent was obtained from the guardian(s). Following the protocol at our center, the fosfluconazole dose was set at 3 mg/kg/dose. The dosing intervals were once every 72 h until postnatal week 2, once every 48 h from postnatal weeks 2 to 4, and daily from postnatal weeks 4 to 6, according to the standard dosing regimen for intravenous fluconazole [28]. The methodology developed was applied to a series of serum samples collected from ten subjects, including neonates receiving fosfluconazole for fungal infection prophylaxis. Serum samples were collected from an arterial line if available. Alternatively, heel-stick blood samples were used. No intervention was done during sampling, and only the samples left over from biochemical examinations were scavenged. The serum samples were collected and stored at − 80 °C for a maximum period of 3 months until processing.

## Results

### Fluconazole detection

**Fig. 1:**
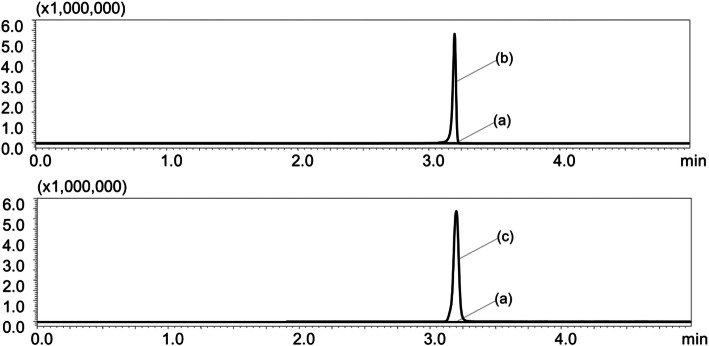
Chromatograms of fluconazole and fluconazole-d4. Chromatograms of fluconazole (upper column) and fluconazole-d4 (internal standard, lower column) in a blank serum sample (**a**) and a spiked sample at a serum concentration of 2 μg/mL (**b**, **c**)

Figure 1 shows chromatograms of a blank serum sample (a) and a serum sample spiked with fluconazole and IS at a concentration of 2.0 μg/mL (b and c). The retention time was approximately 3.2 min for both fluconazole and IS. The total run time for each sample was 5.0 min.

### Linearity, precision and accuracy

**Fig. 2:**
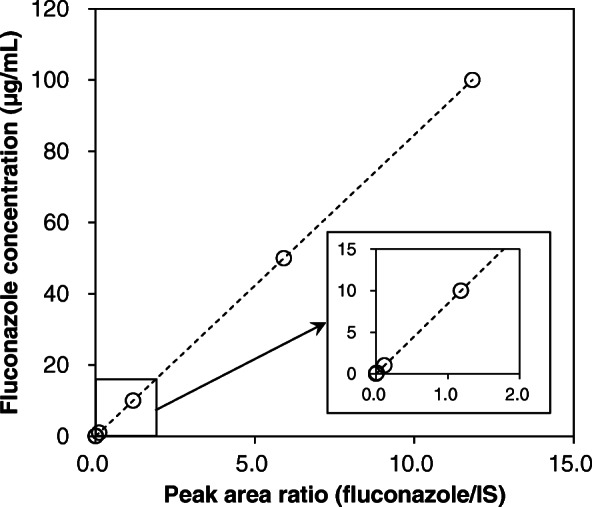
Mean calibration curve for the LC-MS/MS analysis of fluconazole. The horizontal axis indicated the ratio of fluconazole to the internal standard (IS, fluconazole-d4) peak area. The vertical axis indicated the corresponding fluconazole concentrations. The dotted lines indicated the regression lines

Six-point calibration curve was found to be linear over the concentration range of 0.01–100 μg/mL. After comparing the two weighting models (1/x and 1/x^2^), a regression equation with a weighting factor of 1/x^2^ of the drug to the IS concentration was found to produce the best fit for the concentration–detector response relationship for both the analytes in serum samples. Mean equation of calibration curve was y = 8.457x + 0.0006 (Fig. 2), and the mean correlation coefficient of the weighted calibration curves generated during the validation was ≥0.99. Intra and interday accuracy (− 7.7 to 7.4%) and precision (0.3 to 6.0%) were below 15% (Table 2). The estimated LOD by using the residual of standard deviation of regression and the slope of calibration curve was 0.0019 μg/mL while the LLOQ was 0.0031 μg/mL.

**Table 2:** Intraday and interday assay precision and accuracy for fluconazole in serum samples

Conditions	Concentration (μg/mL)	Accuracy (%)	Precision (%)
**Intraday**	0.01	−4.6	3.1
	0.1	4.7	4.6
	1.0	−0.4	2.1
	10.0	7.4	3.4
	50.0	5.0	6.0
	100.0	−7.7	5.8
**Interday**	0.01	−4.4	4.3
	0.1	0.8	2.7
	1.0	3.0	0.3
	10.0	3.2	2.5
	50.0	−2.5	2.5
	100.0	−0.3	5.4

### Recovery

Higher precipitation recovery (98.4 ± 1.9%) was observed after sample preparation than in freshly prepared fluconazole in serum, suggesting that high extraction efficiency ensured fluconazole stability. Specificity testing was performed with blank serum samples from neonates prepared in the same way as the study samples. The endogenous components in the serum samples did not interfere with the compounds of interest.

### Matrix and carry-over effects

The average matrix effects in all the six batches of serum for fluconazole calculated as the response of the post spiked sample/response of neat sample at each two concentration was 0.90 and 0.88, respectively, which indicated negligible significant suppression or enhancement. The variability of matrix effect at each level, as measured by the coefficient of variation, reached ≤15%, thus explaining the reproducible results. The carry-over effect was tested by alternately injecting the highest QC sample and blank samples, and no carry-over was detected.

### Stability

Fluconazole samples were stable during sample preparation and storage. The percentage (mean, relative standard deviation) of remaining fluconazole in the QC samples stored at − 20 °C and − 80 °C for 4 months and that of the freshly prepared samples was 97.7 ± 2.0% and 98.1 ± 1.9%, respectively. In all the QC samples, more than 98.0% of the fluconazole remained after sample treatment at 10 °C for 48 h, indicating that fluconazole was stable for over 48 h in the auto-sampler. The lower percentage of fluconazole remaining in the serum samples stored at room temperature (92.3 ± 3.9%), indicated that the corrected serum samples required storage at − 20 °C or − 80 °C. In freeze and thaw tests, repeated freeze-thaw cycles at six cycles were found to be within ±15% of the predicted concentrations for fluconazole at each five QC levels.

### Application to pharmacokinetic studies

The method validated in this study was applied to 234 serum samples taken from ten neonates who received fosfluconazole for antifungal prophylaxis. The calibration curve with six data points covering a concentration range of 0.01 to 100 μg/mL was used to determination of fluconazole concentration in serum samples. Summary statistics of the patient background, fosfluconazole dosage, and the fluconazole serum concentration after intravenous administration are presented in Table 3. Median time after dose (range) and median serum fluconazole concentrations (range) was 43.7 (0.5–479.7) hours and 2.8 (< 0.01–26.8) μg/mL for the sample from an arterial line, and 40.0 (0.4–467.4) hours and 3.3 (< 0.01–25.9) for the sample from a heel-stick, respectively. Additionally, the median (range) concentration up to 72 h after fosfluconazole administration was 2.9 (0.02 to 26.8 μg/mL) μg/mL. These detected concentrations were within the range of the calibration curve, suggesting that the current method can be used successfully to measure fluconazole levels in neonates. In addition, the concentration-time data can be used to develop a population PK model that can be used in fosfluconazole dose optimization studies of preterm infants.

**Table 3:** Summary statistics of fluconazole serum concentrations after intravenous fosfluconazole administration

Parameters		Value
Number of neonates (male/female)		10 (6/4)
Number of scavenged sampling points		234
Sample volume	< 25 μL	208
	≥ 25 μL	26
Median gestational age (weeks, range)		28.5 (23.3–33.4)
Median weight (g, range)		765 (470–1000)
Median height (cm, range)		33.0 (28.0–38.0)
Median fluconazole dose (mg/kg/dose, range)		3.1 (2.7–3.7)
Sample from arterial lines (*n* = 178)	Median time after dose (hour, range)	43.7 (0.5–479.7)
	Median concentration (μg/mL, range)	2.8 (< 0.01–26.8)
Sample from heel-sticks (*n* = 56)	Median time after dose (hour, range)	40.0 (0.4–467.4)
	Median concentration (μg/mL, range)	3.3 (< 0.01–25.9)

## Discussion

The present study described the development of a selective and sensitive LC-MS/MS method for analyzing fluconazole in serum samples from preterm neonates receiving fosfluconazole. This method allowed a reduction in the serum sample volume to 10 μL, which is a realistic residual serum sample volume in real-world daily practice, and which enabled reliable quantification of fluconazole in blood samples obtained from preterm neonates. Acetonitrile precipitation is straightforward and does not require complicated liquid-liquid extraction or a costly solid-phase extraction column with a large amount of organic solvent. Our methods in this study have no novelty except for the initial amount of sample and sample pretreatment, and no specific techniques was used. However, developing the valid analytical methods contributes to improve the feasibility of pharmacokinetic analysis in preterm neonates. Although the data about fluconazole levels after fosfluconazole treatment in preterm neonates is limited, the detected serum fluconazole concentration was comparable levels in premature neonates who were treated with 4.5 to 6 mg/kg of fluconazole injection [29, 30]. One of the limitations of this study was that the determination of fluconazole from plasma samples was not done. To increase feasibility by using scavenged samples for neonates, assay validation by using both serum and plasma samples is desirable. Additionally, verifying whether there is any difference in fluconazole concentration depending on the sampling site (sampling from an arterial line or a heel-stick blood sampling) is required.

## Conclusion

LC-MS/MS detection method of quantifying fluconazole in neonates using a 10 μL serum sample developed. This method may enable reliable quantification of fluconazole in neonatal blood samples for PK studies without the need for the larger sample quantities required by conventional methods.

## Data Availability

Not applicable.

## Abbreviations

VLBW: Very low birth weight
ELBW: Extremely low birth weight
NICU: Neonatal intensive care unit
PK: Pharmacokinetic
LC-MS/MS: Liquid chromatography-tandem mass spectrometry
IS: Internal standard
QC: Quality control
LOD: Limit of detection
LLOQ: Lower limit of quantification
UV: Ultraviolet
PP: Protein precipitation
LLE: Liquid-liquid extraction
SPE: Solid phase extraction
DBS: Dried blood spot
